# Evaluation of a standardized treatment regimen of anti-tuberculosis drugs for patients with multi-drug-resistant tuberculosis (STREAM): study protocol for a randomized controlled trial

**DOI:** 10.1186/1745-6215-15-353

**Published:** 2014-09-09

**Authors:** Andrew J Nunn, ID Rusen, Armand Van Deun, Gabriela Torrea, Patrick PJ Phillips, Chen-Yuan Chiang, S Bertel Squire, Jason Madan, Sarah K Meredith

**Affiliations:** Medical Research Council Clinical Trials Unit at University College London, Institute of Clinical Trials & Methodology, Aviation House, 125 Kingsway, London, WC2B 6NH UK; International Union Against Tuberculosis and Lung Disease, 68, bd Saint-Michel, 75006 Paris, France; Institute of Tropical Medicine, Nationalestraat 155, 2000 Antwerp, Belgium; Division of Pulmonary Medicine, Department of Internal Medicine, Wan Fang Hospital, Taipei Medical University, No 111, Section 3, Hsin-Long Road, Taipei City, 116 Taiwan; Centre for Applied Health Research & Delivery, Liverpool School of Tropical Medicine, Pembroke Place, Liverpool L3 5QA UK; Warwick Medical School, University of Warwick, Gibbet Hill Road, Coventry, CV4 7AL UK

**Keywords:** Multi-drug-resistant tuberculosis, Multicenter randomized trial, Non-inferiority, Shorter treatment duration

## Abstract

**Background:**

In contrast to drug-sensitive tuberculosis, the guidelines for the treatment of multi-drug-resistant tuberculosis (MDR-TB) have a very poor evidence base; current recommendations, based on expert opinion, are that patients should be treated for a minimum of 20 months. A series of cohort studies conducted in Bangladesh identified a nine-month regimen with very promising results. There is a need to evaluate this regimen in comparison with the currently recommended regimen in a randomized controlled trial in a variety of settings, including patients with HIV-coinfection.

**Methods/Design:**

STREAM is a multi-centre randomized trial of non-inferiority design comparing a nine-month regimen to the treatment currently recommended by the World Health Organization in patients with MDR pulmonary TB with no evidence on line probe assay of fluoroquinolone or kanamycin resistance. The nine-month regimen includes clofazimine and high-dose moxifloxacin and can be extended to 11 months in the event of delay in smear conversion. The primary outcome is based on the bacteriological status of the patients at 27 months post-randomization. Based on the assumption that the nine-month regimen will be slightly more effective than the control regimen and, given a 10% margin of non-inferiority, a total of 400 patients are required to be enrolled. Health economics data are being collected on all patients in selected sites.

**Discussion:**

The results from the study in Bangladesh and cohorts in progress elsewhere are encouraging, but for this regimen to be recommended more widely than in a research setting, robust evidence is needed from a randomized clinical trial. Results from the STREAM trial together with data from ongoing cohorts should provide the evidence necessary to revise current recommendations for the treatment for MDR-TB.

**Trial registration:**

This trial was registered with clincaltrials.gov (registration number: ISRCTN78372190) on 14 October 2010.

## Introduction

Tuberculosis (TB) that is sensitive to standard drugs is a curable disease; most patients can be treated effectively and inexpensively in six months with a regimen that is based on rifampicin and isoniazid. However, multi-drug-resistant TB (MDR-TB), a disease that is resistant to isoniazid and rifampicin, is much more difficult to treat. Current treatment of MDR-TB is based on more drugs given for a much longer time; it is more toxic, has much less satisfactory outcomes and is more expensive [1, 2]. There were an estimated 450,000 cases of MDR-TB worldwide in 2012, although less than 25% of these were estimated to have been detected, and fewer still treated [1]. The diagnosis-treatment gap may be due, in part, to the absence of an accessible and simple treatment regimen.

In 2011, the World Health Organization (WHO) revised their guidance on the diagnosis and management of drug-resistant TB [3]. The recommended duration of the intensive phase was increased to eight months, and in patients without previous anti-TB treatment a total duration of a minimum of 20 months was recommended. However, in contrast to recommendations for treating drug-sensitive TB, both these recommendations were conditional and were based on ‘very low-quality evidence’, as were the recommendations regarding the composition of the regimen [3].

A study carried out in Bangladesh by Van Deun *et al*. published in 2010 reported good success rates in a cohort of over 200 patients with MDR-TB treated with a standardized regimen given for only nine months [4]. Such a regimen would have considerable advantages over standard practice, however, concerns about its reproducibility and generalizability have limited its uptake. In light of this, the STREAM trial was set up with the objective of evaluating the nine-month regimen in a randomized controlled trial to determine whether these promising results could be replicated in other settings, and to compare outcomes to those obtained with the WHO recommended regimen.

This paper describes the design of the STREAM trial, explaining the background and rationale.

## Background

### The nine-month regimen studied in Bangladesh

In recent years a number of meta-analyses of the outcomes of MDR-TB treatment have been published. In a paper by Orenstein *et al*. 34 clinical reports with a mean of 250 patients per report were included in the analysis [5]. Individualized treatment regimens had a summary success rate of 64% (95% CI 59 to 68%) defined as cure or treatment completion; standardized regimens had a summary success rate of 54% (95% CI 43 to 68%) [5]. A more recent systematic review found similar results [2], although data published by WHO suggest that the true success rate for the patients treated with standardized regimens may well be less than 50% [1].

A prospective observational study conducted over a 12-year period in Bangladesh reported on six successive cohorts of patients treated for the first time with a standardized regimen for MDR-TB [4]. The first regimen was largely based on the 1996 WHO guidelines [6]. Periodic assessment of treatment outcomes, relapses and adverse drug reactions was used to guide the introduction of changes for subsequent cohorts. The intention was to develop an effective, safe and inexpensive treatment regimen. The sixth treatment regimen proved to be the most effective; a cohort of 206 patients was treated for a total of 9 to 12 months, the duration of the intensive phase being dependent on response at four months on smear microscopy. Patients were treated throughout with high-dose gatifloxacin, clofazimine, ethambutol and pyrazinamide supplemented by prothionamide, kanamycin and double-dose isoniazid during the four-month intensive phase. The relapse-free cure rate was 87.9% (95% CI 82.7 to 91.6) [4].

The Bangladesh project targeted maximum effectiveness rather than efficacy. Drugs such as PAS (4-aminosalicylic acid) and cycloserine, whose activity is offset by toxicity and poor tolerability, were therefore avoided, and other more active drugs that are also difficult to tolerate, such as prothionamide, were limited to the intensive phase [7]. These were added to a foundation of selected first-line drugs that might be expected to work in at least some patients: ethambutol, isoniazid and pyrazinamide. High doses of selected drugs were used in an effort to maximize effectiveness. In an experimental model it has been shown that selection of resistant mutants could be avoided by using high-dose moxifloxacin [8]. A moderately high isoniazid dose was chosen to overcome at least the low-level resistance conferred by the *inhA* mutation causing cross-resistance to the thioamides, without provoking excessive adverse effects. Inclusion of clofazimine in the regimen as a potentially effective drug was based on *in vitro* testing, although it has never been properly assessed in humans, and cell and animal models have yielded contradictory results. Additional arguments for inclusion of clofazimine were its lack of known serious toxicity from long-term use for leprosy, and a possible synergy with isoniazid [9].

An additional over-riding objective was to avoid amplification of resistance and the creation of XDR-TB; XDR-TB is defined as TB that has developed resistance to rifampicin and isoniazid as well as to any member of the quinolone family and at least one of the following second-line anti-TB injectable drugs: kanamycin, capreomycin, or amikacin. It was felt that prolonged use of less potent drugs would have this effect in cases failing to respond to the core drugs driving the regimen. The regimen appears to have been successful in this regard; only one case of XDR was created in a strain initially susceptible to injectables and clofazimine among over 500 patients enrolled between 2005 and 2011 in Bangladesh [10, 11]. High cure rates of MDR-TB were achievable only with the introduction of fluoroquinolones [12]. The second-line injectables were thus not considered as core drugs, but only as powerful companion drugs protecting the fluoroquinolones. When they fail to do so within the first months of treatment, resistance will be acquired, first to the fluoroquinolones and subsequently to second-line drugs as well. Moreover, limiting their duration to the point of smear-conversion or early declaration of treatment failure would also limit their dose-dependent ototoxicity. The same reasoning applied to the thioamides (prothionamide and ethionamide), and indeed, not a single case of acquired resistance to these drugs was found among the Bangladesh recurrences.

The treatment duration of the regimen was set at nine months based on the improved sterilizing activity of the fourth generation fluoroquinolones in the mouse model [9], and the total absence of relapse in previous Bangladesh cohorts treated for 15 months with ofloxacin (a drug with an inferior sterilizing capability).

### The need to assess the nine-month Bangladesh regimen in other settings

The promising results from Bangladesh prompted a 2008 external review of the pilot study, initiated by the International Union Against Tuberculosis and Lung Disease (The Union), and led by the World Health Organization (WHO). The external review concluded that additional evidence from larger studies was needed in order to determine whether scale-up of a nine-month regimen could be recommended in less controlled conditions. Additionally, the review concluded that this should preferably be done in randomized controlled clinical trials conducted under the requirements of Good Clinical Practice (GCP). It was also considered important to assess the regimen in the presence of HIV-coinfection. The Union’s response to these recommendations was to mobilize resources to undertake such a clinical trial.

In April, 2008, shortly after the results of the external review were reported by the WHO, the United States Agency for International Development (USAID) released a request for proposals for ‘Tuberculosis Research to Enhance the Prevention, Detection, and Management of TB cases’. A multi-site clinical trial to determine the optimal use of existing drugs for MDR-TB treatment was highlighted as a key component of the research agreement. The Union, with a team of international and regional partners, successfully responded to the request for proposals with the five-year Cooperative Agreement in September, 2008. A priority within the initial work plan of the Agreement was the implementation of a clinical trial to evaluate the Bangladesh regimen. In collaboration with the Medical Research Council Clinical Trials Unit at University College London (United Kingdom), the STREAM trial was designed and initiated. There were a number of important logistical issues to overcome before the trial could start, including determining the most suitable trial design and protocol, procurement of the study regimen medicines and supplies and securing the necessary global- and country-level authorizations and approvals. The trial opened to recruitment in July 2012.

### Primary objectives

The primary objectives of the STREAM trial are two-fold. Firstly, to assess whether the proportion of patients with a favorable efficacy outcome on the nine-month study regimen is not inferior to that on the control (WHO-approved MDR-TB) regimen. Secondly, to compare the proportion of patients who experience a grade 3 or greater adverse event (using the Division of AIDS criteria for grading the severity of adverse events) during treatment or follow-up in the nine-month regimen as compared to the control regimen.

## Methods

### Choice of study regimen and choice of comparator

The intention of the study was to evaluate the safety and efficacy of the Bangladesh regimen in a randomized controlled trial in comparison to the WHO recommended standard of care. The nine-month study regimen consists of moxifloxacin, clofazimine, ethambutol and pyrazinamide given for nine months (40 weeks), supplemented by kanamycin, isoniazid and prothionamide in the first four months (16 weeks). All drugs are given in a single dosage daily (seven days a week) except for kanamycin which is given three times per week from week 12. The intensive phase can be extended from 16 to 20 or 24 weeks for patients whose smear has not converted by 16 or 20 weeks respectively. The control regimen is the locally-used WHO-approved MDR-TB regimen. One modification had to be made to the nine-month regimen described by Van Deun *et al*. [4] due to the limited global availability of gatifloxacin manufactured to Good Manufacturing Practice standards at the time the study was being developed. Alternative fluoroquinolones were all considered and it was decided to employ moxifloxacin on the basis of its similarity to gatifloxacin in terms of bactericidal activity at the same dose [13, 14]. It was recognized that the doses of moxifloxacin which would be used for patients in the higher weight bands were not standard and might carry an increased risk of adverse effects, and that careful safety monitoring would be essential. Table 1 shows the doses of each drug in the nine-month regimen that is being evaluated in the STREAM trial. The replacement of gatifloxacin with moxifloxacin at the same dose is the only modification from the regimen studied in Bangladesh.

**Table 1:** **nine-month study regimen drugs and doses**

Product	Weight group		
	Less than 33 kg	33 to 50 kg	More than 50 kg
Moxifloxacin	400 mg	600 mg	800 mg
Clofazimine	50 mg	100 mg	100 mg
Ethambutol	800 mg	800 mg	1200 mg
Pyrazinamide	1000 mg	1500 mg	2000 mg
Isoniazid*	300 mg	400 mg	600 mg
Prothionamide*	250 mg	500 mg	750 mg
Kanamycin*	15 mg per kilogram body weight (maximum 1 g)		

*Given only during the 16-week intensive phase which can be extended to 20 or 24 weeks in the event of delayed smear conversion.

Since the regimen being studied is standardized, in contrast to individualized regimens which are tailored to an individual’s particular drug sensitivity results, it would be most relevant to settings where standardized regimens are used. Therefore, a standardized regimen that followed WHO recommendations was considered an appropriate comparator. WHO recommendations are guidelines which include some latitude in their implementation, resulting in different regimen compositions in different settings. Mandating any one particular standardized regimen as the comparator for the trial would be a departure from standard of care for some treatment programs and therefore not deemed a reasonable comparison. It was therefore decided that the control regimen for the STREAM trial should be the locally mandated standardized regimen in use at the study site provided that it complied with WHO guidelines. This regimen does vary by site, but since randomization in the trial is stratified by site, valid inference can still be made using data from the whole study population.

### Site selection

The selection of sites for the STREAM trial was undertaken in three stages. The initial selection of potential countries followed predefined criteria. These included: sufficiently high overall MDR-TB burden (including both diagnosed MDR-TB cases and estimated MDR-TB cases), a well-functioning basic treatment program (using low default- and low transfer out-rates as proxy measures), the presence of a Green Light Committee (GLC) approved treatment site which would ensure satisfactory control regimen implementation and the presence of TB-HIV treatment activities to ensure adequate management of TB-HIV co-infection within the trial. The GLC is a WHO committee set up to promote access to and rational use of second-line anti-tuberculosis drugs. Furthermore, given the intent for a pragmatic and programmatic-based trial, the entry point for site assessment was the national tuberculosis programs (NTP) of selected countries. Only upon acceptance of the concept by a representative of the NTP and a commitment from the NTP that successful trial results would result in consideration of the regimen for the program was further site assessment and evaluation conducted.

Following a determination of country suitability based on the criteria specified, a site questionnaire was completed by a program representative. Information requested included: number of cases diagnosed and number initiated on treatment in the previous year, patient management practices such as hospitalization and measures to ensure adherence and anticipated proportion of MDR-TB patients who would be eligible for STREAM based on the inclusion and exclusion criteria.

Finally, where the site questionnaire suggested likely suitability for the trial, a site assessment visit was conducted. The site visits assessed capacity to implement a clinical trial, strength of MDR-TB clinical management and laboratory capacity with respect to trial laboratory procedures. Other trial-related components, such as pharmaceutical services and data management facilities were also assessed. The ultimate decision to include a site in the trial was based on the findings of the site assessment visit.

### Patient eligibility criteria

Apart from willingness to participate in the clinical trial, the main eligibility criterion is rifampicin resistance demonstrated by drug susceptibility testing (DST). Acceptable methods of DST for this purpose are line-probe assay (LPA), Xpert MTB/RIF or conventional phenotypic testing. Since patients infected with rifampicin-resistant but isoniazid-sensitive organisms are commonly managed in the same way as patients with MDR-TB and evidence of isoniazid resistance is not necessary for inclusion in the trial. Patients with strains showing resistance to fluoroquinolones and/or second line injectables on rapid LPA are not eligible. While lacking sensitivity [15], it was felt that this test would at least allow exclusion of highly resistant cases in danger of resistance amplification to XDR-TB, without excessively delaying enrolment and start of treatment. Patients in critical condition and those with pre-existing liver disease with aspartate transaminase (AST) or alanine aminotransferase (ALT) greater than five times the upper limit of normal, or corrected QT interval (time between the start of the Q wave and the end of the T wave in the heart’s electrical cycle) of greater than 500 ms, are also excluded because of the limited safety information on the regimen. Premenopausal women are required to use effective barrier contraception or have an intrauterine contraceptive device during the treatment phase and pregnant or breastfeeding women are excluded. All participants are required to have an HIV test and, if positive, to be treated with anti-retroviral treatment (ART) in accordance with the national policies.

### Recruitment process

Potentially eligible patients are referred to study sites for screening to ascertain whether they satisfy the eligibility criteria for enrolment. At their first visit, the study is explained, including the potential risks and benefits associated with participation. Informed consent is obtained before any protocol-specific screening procedures are carried out. Each patient is asked to sign (or provide a thumbprint in the presence of a witness if illiterate) for the screening procedures and is given a copy of the signed consent form and a patient information sheet to take home.

Investigations undertaken include sputum samples for smear, rifampicin resistance testing by LPA or GeneXpert (unless there is a result from another reliable source indicating resistance), LPA for second-line injectables and fluoroquinolone if rifampicin resistant. Blood samples are obtained for HIV antibodies (unless the patient is already known to be HIV positive) and liver function tests (AST and ALT).

Patients are re-assessed for eligibility in the light of the results of the laboratory tests when they return after their screening visit. The time between the screening and enrolment visits is kept as short as logistically possible, but must be no more than four weeks; those returning after four weeks must be re-screened prior to enrolment.

### Treatment allocation

Allocation of treatment for eligible patients is web-based and controlled through an authorized user name and password. Separate randomization lists for each combination of strata (site and HIV status) are prepared in advance by a statistician independent of the study, using varying block sizes. Should web access not be available at the time of randomization, a manual alternative using sealed envelopes is available. The opaque sealed envelopes are kept in a locked box by the local trial pharmacist. Patients are enrolled only after they are found to have satisfied all the enrolment criteria.

### Blinding to treatment assignment

Because of the nature of the trial interventions it is impossible to conduct the trial blind to treatment allocation to the participants, care givers or data managers. All laboratory assessments, including microbiological assessments which form the basis of the patient’s outcome, are conducted blind within both the local and reference laboratories. We accept that local physicians may be influenced in their decision as to whether treatment changes should be made by the knowledge of the treatment allocation. For this reason we have requested that before any changes are made the local physician discusses them with the central medical team (see section below).

### Duration of follow-up and assessment of the primary outcome

One of the major design challenges of the trial was to identify the most appropriate timing for the primary outcome, considering the very different durations of the regimens. In recognition of the fact that patients allocated to the control regimen might receive up to 24 months of treatment it was decided to set the primary outcome as status at 27 months post-randomization, which is very soon after the end of treatment for the control regimen but 18 months after the end of the study regimen. This could potentially result in bias in favor of the control regimen since there is less time after the end of treatment for patients to relapse. Follow-up is every four weeks throughout the trial for patients on both arms. At each assessment sputum samples are collected for smear and culture examination. Positive isolates are sent to the study reference laboratory, the Institute of Tropical Medicine in Antwerp (Belgium), for drug sensitivity testing and strain typing for distinguishing relapses from reinfections.

A single isolated positive culture detected in a clinical trial cannot be assumed to be indicative of treatment failure or relapse [16]. While there is much experience of defining failure and relapse in trials of drug sensitive TB [17], there is no such clear guidance for trials in MDR-TB, and often the treating clinician is best placed to determine whether a patient is failing treatment or relapsing based on a combination of clinical and microbiological data. For this reason, the definition of the primary outcome is driven not only by the culture results, but also by changes to, or restarting of treatment.

Any of the following constitute an unfavorable outcome: positive culture results at 27 months, a change of two or more drugs in the assigned regimen or extension beyond replacement of any missed treatment, restarting treatment or death from any cause during treatment or follow-up. Furthermore, patients who are lost to follow-up before 15 months post-randomization will be considered to have an unfavorable outcome. Those lost after 15 months whose two previous culture results were negative will be considered not assessable in the primary analysis and unfavorable otherwise. Patients who are culture-negative at 27 months will be classified as having a favorable outcome unless they have been otherwise classified as unfavorable.

### Sample size assumptions

The objective of assessing the nine-month regimen in a range of settings could have been addressed in a number of different ways. One way would have been to prospectively enroll further observational cohorts in a number of different countries and report on outcomes. However, unlike the standard six-month regimen for drug-sensitive TB which has been extensively studied in clinical trials in a wide variety of settings with consistent results [15], there is a paucity of data from controlled trials or well-conducted cohort studies of treatment for MDR-TB from which a relatively accurate estimate could be derived to serve as an external comparator; besides which results from observational cohorts are often difficult to evaluate because of selection biases. The alternative for providing high-quality evidence for the efficacy and safety of the nine-month regimen was to conduct a randomized controlled trial in which patients were to receive either the nine-month regimen or the WHO recommended standardized regimen, and this was therefore the approach that was chosen for the STREAM trial.

Currently reported treatment outcomes of MDR-TB are unsatisfactory [1]. If it were to be assumed that the control regimen was 65% effective and the nine-month regimen 85% effective, similar to what was found in Bangladesh, fewer than 220 patients would be required to demonstrate superiority with 90% power. These assumptions, however, are likely to be unrealistic under trial conditions since the standardized regimen is likely to perform better than the average success rate reported in the systematic reviews and the Bangladesh regimen might not perform as well outside of Bangladesh. Using more realistic assumptions of 70% favorable outcomes for the control and 75% for the nine-month regimen, the numbers required for a superiority comparison, even with only 80% power, would be over 1000 per arm, leading to a trial that would take many years to enroll thereby greatly delaying the results. However, even if a nine-month regimen that was much shorter, less expensive with fewer side effects was shown to be no worse (non-inferior) to the standard regimen, this would be a major advance. The STREAM trial therefore has a non-inferiority design with the primary objective being to evaluate whether the nine-month regimen is non-inferior to the WHO standardized regimen.

It is assumed for the power calculations that the nine-month regimen is 5% superior to the control WHO regimen (75% versus 70% favorable outcome). Given a non-inferiority margin of 10% and an assumption that as many as 20% of patients will not be assessable in the primary analysis (see below for discussion of this category), then a total of no more than 400 patients would be required to demonstrate non-inferiority, with 80% power and 5% significance (two-sided). Twice as many patients are allocated to the study regimen as to the control regimen in order to collect more data on the efficacy and safety on this regimen. In the event that non-inferiority were to be demonstrated, it would be appropriate to formally evaluate whether the nine-month regimen was superior, although the power to do so would be low unless the difference was large.

### Analysis of the primary endpoints

For the primary efficacy analysis, the difference in proportion of favorable outcomes between the control regimen and the nine-month regimen with 95% confidence intervals will be estimated. The analysis will be stratified by the randomization stratification factors, site and HIV status. The upper bound of the 95% confidence interval of the difference in proportion favorable between the control and nine-month regimens must be less than 10% (the margin of non-inferiority) in both the modified intention-to-treat (mITT) and the per protocol (PP) populations for the nine-month regimen to be declared non-inferior to the control.

Because of the substantial difference in treatment duration we expect it to be harder to retain patients receiving the shorter duration regimen for the duration of the follow-up period. This increases the chance that these patients will be classified as unfavorable or as unable to be assessed, potentially biasing the results towards failure to declare non-inferiority of the nine-month regimen.

Secondary efficacy analyses include sensitivity analyses to assess the consistency of findings, results in pre-specified subgroups such as HIV-infected patients and an analysis based only on the long-term bacteriological status, irrespective of any changes or modifications of allocated treatment.

The primary safety outcome is the occurrence of grade 3 or greater adverse events as evaluated by the Division of AIDS criteria for grading the severity of adverse events. This analysis will be repeated in subgroups according to HIV infection status and drug resistance patterns.

### The central medical team

First-line anti-tuberculosis drugs are in general well-tolerated with a relatively low frequency of major adverse events. Other drugs used in the treatment of MDR-TB are in general more toxic than those given for drug-sensitive TB. A very high proportion of MDR-TB patients (up to 70% in several reports) on second-line anti-tuberculosis drugs experience one or more adverse drug reactions [18, 19], the management of which is one of the most challenging aspects of MDR-TB treatment. Although most of these side effects are relatively minor, some are serious, either life-threatening or potentially resulting in damage to vital organs (such as hepatitis, renal toxicity, optic neuritis, ototoxicity, severe neurological or psychiatric disturbance), and may require that the responsible drugs are permanently removed from the regimen. To date, there have been few clinical trials that provide information to guide the management of patients with adverse drug reactions in MDR-TB. Thus, the evidence base for guidelines to assist the clinical management of patients who experience adverse drug reaction is lacking. In most cases, management relies on clinical judgment and experience, balancing the risks of continuing toxic treatment compared to the risks associated with the use of less efficacious drugs. The clinician has to try to judge whether it is better to interrupt treatment temporarily to safeguard patient safety, whether it is safe to re-introduce potential toxic drugs after temporary interruption of treatment, how to select the optimal timing and sequence in the re-establishment of an effective regimen and whether it is necessary to add new drugs if one or more drugs are withdrawn from the treatment regimen. If healthcare workers are not familiar with, or not well-trained in the management of adverse effects of the anti-tuberculosis drugs, they may discontinue treatment unnecessarily, re-introduce drugs with suboptimal timing, add new drugs to the treatment regimen unnecessarily or terminate treatment prematurely.

As treatment change is closely linked to outcome assessment in the STREAM trial, and clinicians in trial sites may have different experience and approaches in the management of patients with adverse drug reactions, it was decided to establish a central medical team to provide clinical guidance on the management of patients with adverse drug events to promote consistent management across sites.

The central medical team is an international panel with expertise in MDR-TB treatment, antiretroviral therapy, clinical microbiology and electrocardiology. The panel works remotely, communicates primarily by email with occasional face-to-face meetings and is coordinated by the clinical co-chief investigator. Site investigators may request advice on any clinical issues, but are asked to inform the central clinical team if they think it may be necessary to make a change to the treatment regimen the patient is receiving. The co-chief investigator will request the central medical team to provide input on how to manage the patients. The central medical team is asked to provide comments in a timely manner (usually within 1 to 2 days) to ensure efficiency in the management of patients with adverse drug events. The coordinator integrates the expert advice of the central medical team and forwards it to the site principal investigator. However, it is purely advice and the decisions about clinical management of the patients remain with the site principal investigator. Communication between trial sites, the co-principal investigator and the central medical team continues till the problem is resolved.

### QT monitoring and the management of QT prolongation

The inclusion in the STREAM regimen of high doses of moxifloxacin, which is known to cause QT prolongation [20], together with the fact that many of the patients would also be taking antiretroviral medication which might also impact QT, made the careful monitoring of the cardiac effects of the regimen essential. The protocol requires each patient to have a 12-lead electrocardiogram (ECG) prior to randomization, and anyone with a corrected QT interval (QTc) of 500 ms or greater is not eligible for the study. Further 12-lead ECGs are undertaken at two and four hours after the first treatment dose, then weekly for the first four weeks and at the end of weeks 12, 24 and 36. This was subsequently changed to four-weekly for one year. A 24-hour Holter ECG is undertaken the first time the 12-lead QTc is ≥450 ms (the upper limit of normal for men and below the upper limit of normal for women [21, 22]) to identify diurnal variation which takes the QTc over 500 ms. If the QTc is confirmed as being over 500 ms the cause is sought, and if the patient is on high-dose moxifloxacin the dose is adjusted or levofloxacin substituted to bring the QTc to <500 ms.

Sites are provided with standardized ECG equipment with automatic reporting facilities and a cardiologist is a member of the central medical team and is available to review ECGs of concern. An independent data monitoring committee (IDMC) including two infectious diseases clinicians, a cardiologist and two statisticians has been formed which reviews efficacy and safety data at six-monthly intervals including data on QT prolongation.

### Health economic assessment

The diagnosis and treatment of TB imposes a substantial economic burden on both health systems and patients. This is of particular concern given the prevalence of TB in countries with limited resources, and among the poorest populations in those countries. The economic burden of MDR-TB is particularly acute, given the duration and intensity of treatment. Even when treatment is free, patients face substantial direct and indirect costs when accessing TB services [23]. This financial burden has been linked with poor adherence, and may have severe long-term implications for the financial security of patients and their families, particularly among the highly disadvantaged groups who form a large proportion of TB patients.

Shorter treatment regimens have the potential to reduce the burden of MDR-TB treatment on patients and health systems. However, the impact will depend on factors such as adverse events and treatment success rates. The economic impact of shorter treatment regimens will also depend on the timing of patient and health-system costs; they will have a greater impact if costs are evenly distributed rather than ‘front-loaded’ [24]. An understanding of the health economics of alternative MDR-TB regimens is therefore required by policymakers planning TB services and social protection programs for TB patients. With this in mind, collection of data on health system resource use and patient costs has been incorporated into the STREAM study design.

Every patient recruited into selected sites of the main study is asked to participate in the health economic component of the study. Data are collected on costs incurred in adhering to the regimen, as well as those related to the management of adverse events. Data are collected at recruitment and every three months thereafter, and includes socioeconomic status, education, employment, housing and ownership of physical assets and the costs of food, transport and medications.

The impact of alternative regimens on healthcare resource use are being assessed by identifying the processes and activities involved in the delivery of each regimen, and the management of adverse events. The resources used in staff time, consumables and fixed assets are identified and measured. Unit costs are collected to allow calculation of the total health system cost for each regimen. Data are collected by interviewing appropriate personnel and from records held by funders and healthcare providers.

### Ethical approval

The trial received ethical approval from the Ethics Advisory Group of the International Union Against Tuberculosis and Lung Disease (approval number 07/11) and from the ethics committees of each of the participating sites, namely: South African Medical Research Ethics Committee (approval number EC011-8/2011), Wits Health Consortium Protocol Review Committee (approval number 110602), the Ethics Committee of the Socialist Republic of Vietnam (approval number 661/QD-BYT), St Peter TB Specialized Hospital Ethical Review Committee, Addis Ababa and AHRI-ALERT Ethical Review Committee, Addis Ababa (approval number PO 23/11).

### Dissemination of trial findings

Results of the trial will be published in peer-reviewed journals in accordance with the Consort 2010 statement including the extension for non-inferiority trials [25, 26]. Findings will be presented at international and national conferences, shared with local health ministries and with departments of WHO and other international organizations responsible for producing guidelines for the treatment of MDR-TB.

## Discussion

In 1971 Cochrane gave TB the accolade of having the best evidence base for treatment of any disease and went on to say that the way in which new treatments for TB were introduced could serve as a model for all new treatments in future [27]. Unfortunately this can no longer be said to be true for TB and certainly not for MDR-TB. The WHO readily admit to the very low quality of evidence underpinning their guidelines for treating MDR-TB because of the complete lack of late-stage randomized clinical trials [3].

The research program conducted in Bangladesh by the Damien Foundation provides excellent systematically collected data for the effectiveness of a series of regimens studied not in a randomized design but in consecutively recruited cohorts [4]. The results obtained from the sixth cohort of over 200 patients were impressive, but opinion was divided as to the impact of potential biases that are always present in observational studies and whether these results could be replicated in different settings. The STREAM trial is evaluating, in a randomized controlled non-inferiority trial, whether the nine-month regimen can be considered to be at least as good, if not better, than the regimen recommended by WHO. Because of the long duration of the latter regimen of 20 to 24 months, and because enrolment to STREAM is not expected to complete before the end of 2014, it will inevitably be several years before the outcome of the trial is known. In the meantime additional cohorts assessing a regimen the same or similar to that studied in Bangladesh are being enrolled in several counties, mainly from sub-Saharan Africa [28, 29]. One advantage of this parallel assessment will be that when the trial results are reported there will be additional data from studies conducted under program conditions.

Phase III clinical trials in MDR-TB take many years to conduct and are expensive. Without good surrogate markers, however, they remain necessary to provide reliable evidence to inform policymakers and give confidence to their recommendations, although credit must be given to the Bangladesh study for identifying the nine-month regimen and generating the hypotheses that are being critically evaluated in the STREAM trial.

## Trial status

Recruitment began at the first site in July 2012 and is expected to be completed by early 2015. Discussions are taking place about the possibility of adding additional arms to a second stage of the STREAM trial.

## Abbreviations

ART: anti-retroviral treatment
CI: Confidence interval
ECG: Electrocardiogram
GCP: Good clinical practice
GLC: Green Light Committee
HIV: Human immunodeficiency virus
IDMC: Independent data monitoring committee
LPA: Line-probe assay
MDR-TB: Multi-drug-resistant tuberculosis
MRC: Medical research council
NTP: National tuberculosis programs
PAS: para*-*aminosalicylic acid
QTc: Corrected QT interval
TB: Tuberculosis
The Union: International union against tuberculosis & lung disease
UCL: University College London
WHO: World health Organization.
